# Self-reported sleep quality and mental health mediate the relationship between chronic diseases and suicidal ideation among Chinese medical students

**DOI:** 10.1038/s41598-022-23207-8

**Published:** 2022-11-06

**Authors:** Zhen Gui, Long Sun, Chengchao Zhou

**Affiliations:** 1 Centre for Health Management and Policy Research, School of Public Health, Cheeloo College of Medicine, Shandong University, 44 Wenhuaxi Road, Jinan, 250012 Shandong China; 2 National Health Commission of China (NHC) Key Lab of Health Economics and Policy Research (Shandong University), Jinan, 250012 China; 3 Center for Suicide Prevention Research, Shandong University, Jinan, 250012 China

**Keywords:** Psychology, Health care

## Abstract

High prevalence and strong associations of chronic disease, poor sleep quality, poor mental health, and suicidal ideation (SI) have been reported worldwide. However, the underlying mechanism remains unexamined. The participants were 2646 Chinese medical college students with an average age of 20.13 years. Pittsburgh Sleep Quality Index, the Kessler Psychological Distress Scale, and SI was evaluated. The lifetime SI, past 12-month SI, and chronic disease prevalence rates were 10.0%, 8.4%, and 4.6%, respectively. The results of logistic regression analysis in this study found that chronic disease, self-reported poor sleep quality, poor mental health, gender and scholarship were associated with lifetime SI. Similar results were also found for 12-month SI with an exception of region. This result indicated that the effects of chronic diseases on the SI were mediated by self-reported sleep quality and mental health. Physical diseases, sleep-related concerns, and mental health issues need to be addressed through a multidisciplinary team approach and various delivery systems to prevent SI among medical college students.

## Introduction

Suicide accounted for 1.4% of all global deaths in 2016 and was the second leading cause of death for people aged 15–29 years worldwide^1^. In China, it is the fifth most common form of death among the total population (3.6%) and the most prominent cause of death among those aged 15–34 years (19%)^2^. According to a large-scale online survey by the National Alliance for Research, a higher education counseling center, 18% of college students had seriously considered attempting suicide previously, while 6% had deliberated on the same in the past 12 months^3^. In prior studies, the lifetime and past 12-month suicidal ideation (SI) rates among medical students were approximately 18% and 11%, respectively, which were higher than those among other undergraduates^4,5^. Previous studies have also shown that adolescent suicidal ideation as predictive of psychopathology, compromised functioning, and suicidal behavior^6,7^; moreover, it is a considerable risk element for suicide behavior among undergraduates^8^. Therefore, recognizing its risk factors is critical for preventing suicide among medical students.

The full name of chronic disease is chronic non-communicable diseasese, which does not refer to a particular disease, but is a general term for a class of diseases with hidden onset, long course and prolonged illness, lack of exact evidence of infectious biological etiology, complicated etiology, and some diseases that have not been fully identified. The World Health Organization has stated that chronic non-communicable diseasese include cardiovascular diseases, diabetes, cancers, and chronic respiratory ailments^9^. Chronic diseases remain undisputed as the predominant challenge to global health and constitute for nearly two-thirds of the global deaths. Correspondingly, they are also the leading cause of poor health, disability, and death, thus accounting for most health care expenditures^10–12^. In a review, Greydanus suggested an association between chronic illnesses and suicide risk in adolescents^13^. Furthermore, the former has been found to increase the possibility of SI and suicide attempts^14,15^; identical results have been reported among medical students^16,17^. Although a relationship between chronic diseases and SI has been indicated, knowledge of the potential factors and mechanisms underlying it is inadequate. To facilitate the early detection and prevention of SI, we should devote ourselves to explore the possible mediating pathways between chronic diseases and SI, and provide the essential theoretical framework for suicide prevention among medical students from the perspective of public health and clinical work.

Previous studies have indicated that depression, anxiety, stress, and psychological strains are positively related to SI in both Chinese and American college students^18^. Further, mental distress was significantly associated with SI among medical students^19^. In a review, O’Connor and Nock emphasized the key prominence of psychological factors in suicidal thoughts and behaviors^20^. Furthermore, several studies of patients with chronic diseases have reported a significant relationship between mental health and SI beyond the general population^21,22^. Specifically, among adults diagnosed with diabetes, the prevalence of serious mental health problems was twice as high as that among those without it^23^. Moreover, a path analysis has demonstrated that mental health acted as a mediator between chronic diseases and SI^24^, and may play a substantial mediating role in the relationship between the two.

Self-reported sleep quality is strongly associated with individual chronic diseases and poor mental health, and increased suicide risk^25^. For example, poor sleep quality has been indicated to elevate the risk of suicide by approximately 34%^26^. Other studies have also demonstrated that while reduced self-reported poor sleep quality is a strong predictor of SI^27^, chronic diseases also play a major role^28^. A study conducted by medical specialists shows that chronic diseases are closely related to poor sleep quality; moreover, good quality of sleep has been observed in those without any such diseases^29^. Other studies have found sleep disturbances to have a mediating effect on the relationship between non-malignant chronic pain and death by suicide^30^. The aforementioned results indicate that medical students with chronic diseases show a higher tendency to self-report inadequate sleep quality which may further contribute to SI. However, empirical studies that directly investigate the mediating role of self-reported sleep quality in the relationship between chronic disease and SI is insufficient.

A study in Nigeria among undergraduates has identified that poor sleep quality was strongly related to poor mental health^31^. Another meta-analysis in Brazil has reported medical students with low sleep quality to be at an increased risk of mental health problems^32^. Furthermore, research conducted at the Massachusetts General Hospital in the U.S. indicated that undergraduates with depressive symptoms and sleep disturbances may experience additionally intense and frequent anxiety and poorer physical functioning^33^. Moreover, structural equation modeling has revealed sleep quality to mediate the impact of traumatic life events on mental health problem and suicidality among undergraduates^34^. Thus, self-reported sleep quality and mental health may play serial mediating roles in the relationship between chronic diseases and SI.

Recently, Joiner's (2005) Interpersonal Psychological Theory of Suicide (IPTS) is an important contribution to understanding suicidality^35^. Suicide ideation and acts typically begin in adolescence^36,37^. Seventeen studies in adolescents specifically tested and interpreted the findings according to IPTS theory^38^. According to the IPTS, suicidal ideation (SI) results from the experience of either thwarted belonging-ness (TB) or perceived burdensomeness (PB) jointly termed interpersonal states. The presence of both of these states results in the active desire to die. Chronic diseases, psychological stress and sleep quality all contribute to the TB and PB to some extent. Moreover, chronic diseases, in which patients are repeatedly exposed to physical pain and psychological stress over long periods of time, are the second key construction of IPTS, and these will also promote the progression of suicidal intent to suicidal behavior, ultimately leading to suicide^39^.

**Figure 1:**
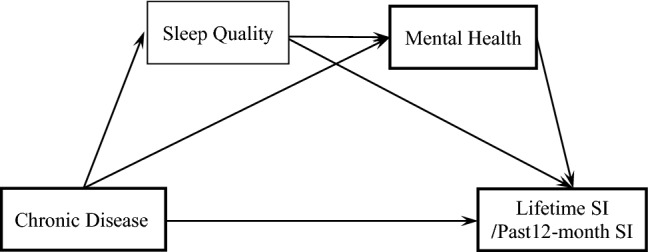
Hypothesized model.

In recent years, studies on the elderly have found that mental health is a mediator of chronic diseases and SI; further, the former and self-reported sleep quality are the two chain mediators on activities of daily living and SI^24,40^. However, thus far, no study has explored the association between chronic disease and among Chinese medical students in a comprehensive and detailed manner, to provide a scientific basis for suicide prevention among medical students. Therefore, this study examined the multiple mediating effects of self-reported sleep quality and mental health on chronic diseases and SI among medical students. We proposed three hypotheses (Fig. 1): first, chronic diseases significantly affect lifetime SI and past 12-month SI in medical students (H1); second, both mental health and sleep quality mediate the relationship between chronic diseases with lifetime SI and past 12-month SI (H2); and third, sleep quality and mental health play a serial mediating role between chronic diseases with lifetime SI and past 12-month SI, respectively (H3).

## Results

### Descriptive analyses

Among all respondents, 10.0% and 8.4% reported the lifetime and the past 12-month SI, respectively. Chronic illness was significantly different from those with or without lifetime SI (*χ*^2^ = 26.85, P < 0.001) as well as the past 12-month SI (*χ*^2^ = 26.85, P < 0.001). Differences in these other factors, such as age, region, family composition, parental relationship, family economic status, and obtain a scholarship, etc., over the past 12-month SI were nearly the same as those in the lifetime SI, with the only exception being grade. Regarding lifetime SI, those experiencing SI (vs. not) reported higher levels of mental health problems (t = 22.11, P < 0.001). Their total score of PSQI was also greater than that of their counterparts (*t* = −10.82, *P* < 0.001) (Table 1).

**Table 1:** Description and univariate analysis of suicidal ideation among the medical college student in Shandong, China (N = 2646).

Characteristics	N (%)	Lifetime suicidal ideation		$\chi$^2^*/t*	Past 12-months suicidal ideation		$\chi$^2^*/t*
		Yes (%)	No (%)		Yes (%)	No (%)	
Observations	2646 (100)	265 (10.0)	2381 (90.0)	–	221 (8.4)	2425 (91.6)	–
Age, years, (mean ± SD)	20.13 ± 1.23	20.27 ± 1.12	20.12 ± 1.24	−1.87	20.19 ± 1.08	20.13 ± 1.24	−0.76
**Gender**				5.16*			7.18**
Male	1174 (44.4)	135 (11.5)	1039 (88.5)		117 (10.0)	1057 (90.0)	
Female	1472 (55.6)	130 (8.8)	1342 (91.2)		104 (7.1)	1368 (92.9)	
**Grade**				4.96*			0.05
Junior	1731 (65.4)	157 (9.1)	1574 (90.9)		143 (8.3)	1588 (91.7)	
Senior	915 (34.6)	108 (11.8)	807 (88.2)		78 (8.5)	837 (91.5)	
**Region**				7.21**			17.43***
Urban	1261 (47.7)	147 (11.7)	1114 (88.3)		135 (10.7)	1126 (89.3)	
Country	1385 (52.3)	118 (8.5)	1267 (91.5)		86 (6.2)	1299 (93.8)	
**Family composition**				3.98*			7.86**
Regular	2439 (92.2)	236 (9.7)	2203 (90.3)		193 (7.9)	2246 (92.1)	
Others	207 (7.8)	29 (14.0)	178 (86.0)		28 (13.5)	179 (86.5)	
**Parental relationship**				13.17***			14.95***
Good	2129 (80.5)	191 (9.0)	1938 (91.0)		156 (7.3)	1973 (92.7)	
Not good	517 (19.5)	74 (14.3)	443 (85.7)		65 (12.6)	452 (87.4)	
**Family economic status**				0.37			0.87
Good	512 (19.3)	55 (10.7)	457 (89.3)		48 (9.4)	464 (90.6)	
Not good	2134 (80.7)	210 (9.8)	1924 (90.2)		173 (8.1)	1961 (91.9)	
**Obtain a scholarship**				11.36***			9.71**
Yes	809 (30.6)	105 (13.0)	704 (87.0)		88 (10.9)	721 (89.1)	
No	1837 (69.4)	160 (8.7)	1677 (91.3)		133 (7.2)	1704 (92.8)	
**Chronic disease**				26.85***			13.12***
Yes	122 (4.6)	29 (23.8)	93 (76.2)		21 (17.2)	101 (82.8)	
No	2524 (95.4)	236 (9.4)	2288 (90.6)		200 (7.9)	2324 (92.1)	
Sleep quality (mean ± SD)	4.95 ± 2.83	6.70 ± 2.92	4.76 ± 2.75	−10.82***	7.00 ± 3.00	4.77 ± 2.74	−11.49***
Mental health (mean ± SD)	19.14 ± 6.65	27.00 ± 6.21	18.26 ± 6.10	−22.11***	27.43 ± 6.28	18.38 ± 6.15	−20.91***

Others family composition include restructured families (67, 2.53%), single-parent families (119, 4.50%) and others (21, 0.79%). *p<0.05; **p<0.01; ***p<0.001.

### Binary logistic regression analysis for the lifetime SI risk among the medical college students

In Table 2, we conducted five models (Models 1 to 5) to identify the relationship between chronic diseases, sleep quality, mental health, and lifetime SI among the medical college students. After controlling for the significant background characteristics, the former three variables were entered step-wise into the logistic regression models. First, chronic diseases and sleep quality were separately added into Models 1 and 2, respectively; the former [odds ratio (OR) = 2.68, 95% confidence interval (CI) 1.71–4.19] and the latter (OR = 1.24, 95% CI 1.19–1.30) were significantly related to lifetime SI. Second, they were collectively included in Model 3; chronic diseases (OR = 2.13, 95% CI 1.34–3.38) and self-reported sleep quality (OR = 1.23, 95% CI 1.18–1.29) were significantly related to the lifetime SI. Third, chronic diseases and mental health were together added to Model 4; the former (OR = 1.70, 95% CI 1.02–2.81) and the latter (OR = 1.22, 95% CI 1.19–1.25) were significantly associated with lifetime SI. Finally, chronic diseases, sleep quality, and mental health were all included in Model 5; we found that for males (OR = 1.36, 95% CI 1.01–1.81), obtaining a scholarship (OR = 1.52, 95% CI 1.12–2.08), diagnosis of a chronic illness (OR = 1.67, 95% CI 1.01–2.77), and higher levels of mental health problem (OR = 1.21, 95% CI 1.18–1.24) were more likely to be related to lifetime SI as compared to their counterparts.

**Table 2:** Binary logistic regression for the factors associated with lifetime suicidal ideation (N = 2646).

	Model 1	Model 2	Model 3	Model 4	Model 5
Age	1.02 (0.88, 1.19)	0.97 (0.83, 1.12)	0.97 (0.84, 1.13)	0.88 (0.75, 1.04)	0.88 (0.75, 1.04)
Male	1.41 (1.09, 1.83)**	1.45 (1.11, 1.90)**	1.46 (1.11, 1.90)**	1.35 (1.01, 1.81)*	1.36 (1.01, 1.81)*
Senior grade (ref. = junior)	1.11 (0.77, 1.60)	1.16 (0.80, 1.67)	1.12 (0.77, 1.62)	1.31 (0.88, 1.95)	1.31 (0.88, 1.95)
Urban region	1.38 (1.06, 1.81)*	1.39 (1.06, 1.83)*	1.41 (1.07, 1.85)*	1.31 (0.97, 1.76)	1.32 (0.98, 1.77)
Regular family composition	0.94 (0.58, 1.50)	1.03 (0.64, 1.68)	1.04 (0.64, 1.69)	0.95 (0.56, 1.62)	0.96 (0.56, 1.64)
Good parental relationship	0.61 (0.44, 0.85)**	0.68 (0.48, 0.95)*	0.70 (0.49, 0.98)*	0.80 (0.55, 1.17)	0.81 (0.55, 1.18)
Good family economic status	1.04 (0.75, 1.46)	1.19 (0.84, 1.67)	1.16 (0.82, 1.64)	1.27 (0.87, 1.85)	1.28 (0.88, 1.86)
Obtain a scholarship	1.48 (1.12, 1.96)**	1.50 (1.13, 1.99)**	1.46 (1.10, 1.95)**	1.53 (1.12, 2.09)**	1.52 (1.12, 2.08)**
Chronic disease	2.68 (1.71, 4.19)***	–	2.13 (1.34, 3.38)***	1.70 (1.02, 2.81)*	1.67 (1.01, 2.77)*
Sleep quality	–	1.24 (1.19, 1.30)***	1.23 (1.18, 1.29)***	–	1.03 (0.97, 1.09)
Mental health	–	–	–	1.22 (1.19, 1.25)***	1.21 (1.18, 1.24)***
Constant	0.06*	0.05*	0.04*	0.01**	0.01**
R^2^	0.043	0.103	0.110	0.303	0.303

Only ORs and their 95% CIs were presented in the table.

*p<0.05; **p<0.01; ***p<0.001.

### Binary logistic regression analysis for the past 12-month SI risk among the medical college students

We adopted five other models (Models 6 to 10) to identify the relationship between chronic diseases, sleep quality, mental health, and the past 12-month SI. The regression analysis methods and procedures for the past 12-month SI were identical to those used in the lifetime SI analysis; additionally, the significant differences in the past 12-month SI were nearly the same as those in the lifetime SI, with the exception of region. Additional details are provided in Table 3. After controlling for the background characteristics, chronic diseases, sleep quality, and mental health were entered into Model 10; subsequently, it was found that in males (OR = 1.49, 95% CI 1.09–2.05), region (OR = 1.76, 95% CI 1.27–2.44), obtaining a scholarship (OR = 1.65, 95% CI 1.17–2.31), poor sleep quality (OR = 1.08, 95% CI 1.02–1.14), and higher levels of mental health problem (OR = 1.20, 95% CI 1.17–1.24) were more likely to be related to the past 12-month SI as compared to their counterparts.

**Table 3:** Binary logistic regression for the factors associated with past 12-month suicidal ideation (N = 2646).

	Model 6	Model 7	Model 8	Model 9	Model 10
Age	1.06 (0.90, 1.24)	0.99 (0.84, 1.17)	0.99 (0.85, 1.17)	0.90 (0.75, 1.08)	0.89 (0.75, 1.07)
Male (ref. = female)	1.52 (1.14, 2.01)**	1.59 (1.19, 2.13)**	1.59 (1.19, 2.13)**	1.47 (1.08, 2.02)*	1.49 (1.09, 2.05)*
Senior grade (ref. = junior)	0.80 (0.53, 1.19)	0.80 (0.53, 1.20)	0.79 (0.53, 1.19)	0.89 (0.58, 1.37)	0.88 (0.57, 1.36)
Urban region (ref. = country)	1.77 (1.32, 2.38)***	1.80 (1.33, 2.44)***	1.81 (1.34, 2.45)***	1.73 (1.25, 2.40)***	1.76 (1.27, 2.44)***
Regular family composition (ref. = others)	0.77 (0.48, 1.26)	0.85 (0.51, 1.41)	0.85 (0.52, 1.42)	0.73 (0.42, 1.28)	0.75 (0.43, 1.31)
Good parental relationship (ref. = not good)	0.58 (0.41, 0.84)**	0.67 (0.46, 0.98)*	0.69 (0.47, 0.99)*	0.77 (0.51, 1.16)	0.80 (0.53, 1.20)
Good family economic status (ref. = not good)	1.05 (0.73, 1.50)	1.21 (0.83, 1.75)	1.19 (0.82, 1.73)	1.29 (0.86, 1.92)	1.32 (0.88, 1.97)
Obtain a scholarship (ref. = no)	1.59 (1.17, 2.15)**	1.60 (1.18, 2.19)**	1.58 (1.15, 2.15)**	1.65 (1.18, 2.32)**	1.65 (1.17, 2.31)**
Chronic disease (ref. = no)	2.17 (1.31, 3.60)**	–	1.63 (0.96, 2.76)	1.30 (0.74, 2.29)	1.24 (0.70, 2.19)
Sleep quality	–	1.28 (1.22, 1.35)***	1.28 (1.22, 1.34)***	–	1.08 (1.02, 1.14)*
Mental health	–	–	–	1.22 (1.19, 1.25)***	1.20 (1.17, 1.24)***
Constant	0.03*	0.02*	0.02*	0.01**	0.01**
R^2^	0.05	0.134	0.136	0.308	0.313

Only ORs and their 95% CIs were presented in the table.

*p<0.05; **p<0.01; ***p<0.001.

### Mediation analyses

The serial multiple mediations of sleep quality and mental health were found to be significant in the relationships of chronic disease with the lifetime and the past 12-month SI. The total, direct, and indirect effects are listed in Table 4.

**Table 4:** The total, direct, and indirect effects of chronic disease on suicidal ideation (N = 2646).

Model pathways	Lifetime suicidal ideation		suicidal ideation within 1 year	
	β	Mediating effect (%)	β	Mediating effect (%)
Direct effect chronic disease → suicidal ideation 95% (LLCI, ULCI)	0.512* (0.005, 0.185)	40.99	0.212 (−0.360, 0.785)	21.33
Total indirect effect chronic disease → suicidal ideation	0.737	59.01	0.782	78.67
chronic disease → sleep quality → suicidal ideation	0.037	2.96	0.101*	10.15
chronic disease → mental health → suicidal ideation	0.401*	32.11	0.390*	39.24
chronic disease → sleep quality → mental health → suicidal ideation	0.299*	23.94	0.291*	29.28

p < 0.05.

### Mediating effect analyses for the lifetime SI

**Figure 2:**
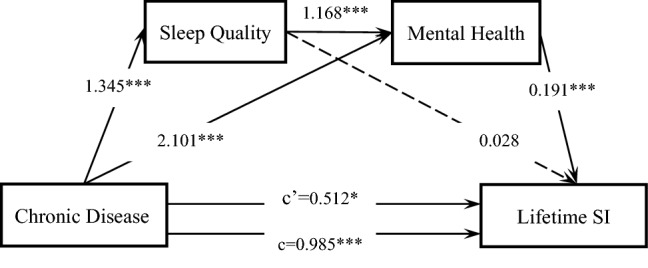
Serial multiple mediation of self-reported sleep quality and mental health in the relationship between chronic disease and lifetime SI. c is the total effect of chronic disease on lifetime SI. c’ is the direct effect of chronic disease on lifetime SI, represented by a dotted line. The other values in the figure are regression coefficients in the model. *p < 0.05, **p < 0.01, ***p < 0.001.

It was observed that chronic diseases had a total (c = 0.985, P < 0.05) and a direct effect on the lifetime SI (c' = 0.512, P < 0.05), after the variables of self-reported sleep quality and mental health were uncontrolled (Fig. 2). Moreover, the indirect effect value of chronic diseases on the lifetime SI through mental health was 0.401, while that through self-reported sleep quality and mental health was 0.299; their mediating effects were 32.11% and 23.94%, respectively. However, there was no statistically significant indirect effect on self-reported sleep quality (P = 0.312). The mediating effect of chronic diseases on lifetime SI was stronger than the direct effect, and the percentage of the mediating effect was 59.01%.

### Mediating effect analyses for the past 12-month SI

**Figure 3:**
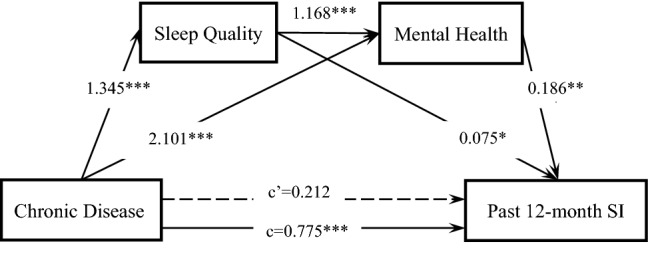
Serial multiple mediation of self-reported sleep quality and mental health in the relationship between chronic disease and past 12-month SI. c is the total effect of chronic disease on past 12-month SI. c’ is the direct effect of chronic disease on past 12-month SI, represented by a dotted line. The other values in the figure are regression coefficients in the model. *p < 0.05, **p < 0.01, ***p < 0.001.

The direct effect of chronic diseases (c' = 0.212, P > 0.05) on the past 12-month SI disappeared after controlling for self-reported sleep quality and mental health (Fig. 3). When considering the mediating variables collectively and separately, a serial multiple mediation of self-reported sleep quality and mental health (β = 0.291; 29.28%), a single mediation of mental health (β = 0.390; 39.24%), and a single mediation of self-reported sleep quality (β = 0.101; 10.15%) were significant. The total mediating effect of chronic diseases on the past 12-month SI was stronger than that of chronic illness on lifetime SI (78.67% and 59.01%, respectively). This result indicated that the effects of chronic diseases on the past 12-month SI were also mediated by self-reported sleep quality and mental health. Additionally, the direct effect of chronic diseases on the past 12-month SI disappeared after self-reported sleep quality was added as the mediator compared with lifetime SI. This might indicate that self-reported sleep quality plays a greater role in mediating chronic diseases with the short-term SI rather than the lifetime SI.

## Discussion

This study found that the prevalence of the lifetime and the past 12-month SI among the medical students in the Shandong Province was 10.0% and 8.4%, respectively. These values were similar to those in previous surveys regarding the past 12-month SI among medical students in the United States and China: 9.4% and 8.2%, respectively^4,41^; however, they were considerably lower than their lifetime SI values: 29.9% and 17.9%, respectively^42^. Given the different levels of research background and the time points of definition, the prevalence of SI varies widely. Furthermore, our major findings were as follows: (1) self-reported sleep quality and mental health played a serial mediating role in the relationship of chronic diseases with the lifetime and the past 12-month SI; (2) self-reported sleep quality mediated the association between chronic diseases and poor mental health; however, (3) after adding self-reported sleep quality as a mediator, the direct effect between chronic disease and the past 12-month SI disappeared; (4) mental health mediated the relationship between chronic diseases and the lifetime SI; and finally, (5) the path through the sole mediation of self-reported sleep quality was non-significant.

In this study, the prevalence of chronic diseases was 4.6%; this was lower than the 10.4% prevalence rate reported by Professor Miauton in a survey of 9,268 in-school adolescents aged 15 to 20 years in Switzerland. The past 12-month SI rates among adolescents with and without chronic diseases were 25.6% and 14.4%, respectively (in this study, 17.2% and 7.9%, respectively; regarding the lifetime SI, 23.8% and 9.4%, respectively). They concluded that suicide attempts are common in young people with chronic illnesses^43^. Similarly, this research found that the prevalence rates of the lifetime and the past 12-month SI among the medical students with chronic diseases were 23.8% and 17.2%, respectively, which were significantly higher than those of healthy students (9.4% and 7.9%, respectively). The former was more vulnerable to SI than the latter. This result was similar to that of another survey on the elderly conducted in rural Shandong that found chronic illnesses to increase the lifetime SI risk (13.2.0% vs. 10.0%)^24^. A previous article on suicidality in chronic illness provided evidence that cognitive, affective, interpersonal, and behavioral factors that may contribute, at least in part, to the association between chronic illness and suicidal thoughts and behaviors^44^.

Previous research indicated that adolescents with chronic diseases are significantly more susceptible to biopsychosocial risk factors; moreover, children with such ailments would have three to four times increased risk of psychiatric problems than their healthy peers^45,46^. Finally, medical students with chronic diseases were more likely to have SI as they experience more physical diseases and mental pressure due to the high burden of emergency rescue. The current significant relationship between chronic disease, self-reported sleep quality, mental health, and SI was confirmed among the findings of this study; the need for additional efforts to monitor chronic diseases and self-reported sleep quality, as well as prevent medical students’ poor mental health to mitigate the risk of SI, is strongly suggested. The significant direct effect between chronic diseases and SI was consistent with previous research^13,24^; this indicated that adolescents with chronic illnesses were more likely to self-report poor quality of sleep and poor mental health, thus leading to negative thoughts and ultimately SI. Nevertheless, the overall mediating effects in the 2 mediation models were 59.01% and 78.67%, both of which were stronger than their direct effects; this reveals that the mediators play a crucial role in accounting for the relationship between chronic illness and lifetime and past 12-month SI.

Our results showed that self-reported sleep quality was a strong mediator of the relationship between chronic illness and mental health in both the SI mediating models (42.8%). This effect was similar to that found in the Chinese elderly, where self-reported sleep quality had a 41.1% mediating effect on the relationship between physical dysfunction and mental health^40^; however, this was a considerably weaker effect than that reported for American college undergraduates, where self-reported sleep quality significantly mediated the negative association between traumatic events, mental health, and suicidality (proportional effects between 10.6 and 12.5%)^34^. Studies on the sleep mechanism have suggested that the brain’s electrophysiological activity during sleep is related to an increased SI; specifically, hyperarousal of the central nervous system during sleep may be the SI’s neurobiological correlate^47^.

Furthermore, we observed that the relationship between chronic diseases and the lifetime SI, as well as the past 12-month SI, in part through a chain of self-reported sleep quality and mental health, showed effect values of 23.94% and 29.28%, respectively. Therefore, this finding supported our hypothesis.

Joiner's (2005) Interpersonal Psychological Theory of Suicide (IPTS) is an important contribution to understanding suicidality^35^. Seventeen studies in adolescents specifically tested and interpreted the findings according to IPTS theory^38^. According to the IPTS, suicidal ideation (SI) results from the experience of either thwarted belonging-ness (TB) or perceived burdensomeness (PB) jointly termed interpersonal states. The presence of both of these states results in the active desire to die. Suicide ideation and acts typically begin in adolescence^36,37^. Chronic diseases contribute to the TB and PB to some extent. Moreover, chronic diseases, in which patients are repeatedly exposed to physical pain and psychological stress over long periods of time, are the second key construction of IPTS, and these will also promote the progression of suicidal intent to suicidal behavior, ultimately leading to suicide^39^. In addition, strain theory also remind us that, chronic diseases, mental health problems and poor sleep quality were associated with suicidal ideation. The Strain Theory of Suicide explains the deep motivation for suicide^48^ and suggests that long-term chronic diseases and treatments may bring inner struggles and pain to medical college students, which in turn produces psychological torsion, thus promoting the formation of SI^49^.

The result that self-reported sleep quality had an impact on mental health was consistent with previous findings that the former was related to the latter^40^; moreover, a mechanism research suggested that sleep quality plays an important and irreplaceable role in the neuro-immuno-endocrine homeostasis. For example, the lymphoid system is associated with neurodegenerative disorders modulated by sleep; further, sleep loss increases the risk of cognitive and mood disorders by impairing synaptic transmissions^50^. These mechanisms are consistent with our study findings that self-reported sleep quality and mental health play a series of mediating roles between chronic diseases and the SI. Collectively, the present findings suggest that chronic illnesses are sequentially and primarily related to poor self-reported sleep quality; subsequently, they cause mental health in medical students, which in turn contributes to SI.

The results from the two mediation models indicated that mental health is an important mediator in the relationship between chronic diseases and the lifetime SI, as well as the past 12-month SI. Therefore, the hypothesis H2 was tested: the medical students with chronic diseases were more likely to develop SI through mental health problems. Mental health factors accounted for 32.11% and 39.24% of the chronic diseases related to the lifetime and the past 12-month SI respectively; this revealed that chronic illnesses moderated the generation of SI mainly through mental health. The positive correlation between mental health and SI has also been established in previous studies^18,22^; if the level of the mental health problem is higher, the possibility of medical students suffering from the SI is higher. Another research demonstrated that chronic diseases prevent SI by improving the mental health among the elderly in China^24^. Mental health plays the most important mediating role in the relationship between chronic illness and SI, which is an important finding of our study. One possible reason is that the former could increase the medical students’ physical pain and financial difficulties, leading to an increase in negative emotions. Chronic diseases or disabilities are clearly responsible for producing the feelings of anxiety and depression^24^. Such people may be perceived differently or even discriminatively by their peers, causing them to feel guilty and useless as well as carry tremendous psychological burden, all of which contribute to the onset of SI.

We also found a strong significant association between obtain a scholarship and SI. Although previous studies have suggested that poor academic performance is a risk factor for SI^51,52^, but most of their studies has focused on students in the lower ages of 16 and under, who themselves are less academically stressed. Medical students who obtain a scholarship are often means better academic performance and more academic stress, which brings mental health problems and leads to the generation of SI. For example, a student whose actual test score is higher than that of other students, but whose test score is lower than the student's own expectations, may still experience a higher risk of suicide. This lower-than-expected academic performance may contribute to psychological tensions, which in turn increases the risk of SI^48^. Specifically, in the high-stakes gaokao, students whose national rankings were lower than expected were more likely to show SI than those who performed better than expected^53^.

Additionally, we found that the path through the sole mediation of self-reported sleep quality was not significant between chronic diseases and lifetime SI. However, self-reported sleep quality accounted for 10.15% of the chronic diseases and the past 12-month SI; furthermore, the direct effect between the chronic illness and past 12-month SI disappeared when self-reported sleep quality was added as a moderator. These findings are also consistent with previous research showing that chronic illnesses can cause severe sleep problems or insomnia^54^ and that poor self-reported sleep quality contributes to the development of SI^25^.

When medical students with chronic conditions self-report inadequate sleep quality, it can lead to a reduction in their daily activities and a shortened sleep–wake cycle. Moreover, Holdaway shows that low sleep quality may increase the risk of various negative thoughts and overall suicide, especially SI.^55^. Previous studies have also shown that sleep quality is a mediator between non-malignant chronic pain and SI, as well as between internet gaming disorder and SI^30,56^.

### Contributions of this study

Medical doctors are a high-risk group for suicide^57^; furthermore, this problem appears to arise with medical students^5^. Chronic diseases pressurize them, and being in contact with suffering, confronting death, and caring for vulnerable persons may be potential threats that trigger negative emotions and poor mental health, ultimately leading to SI^58^. Research on the present situation and the mechanism of the medical students' SI is helpful in drawing the attention of academic, social, school, educational, and government departments to their chronic diseases and mental health. Additionally, this study aimed to explore the serial multiple mediating effects of sleep quality and mental health on the association between chronic diseases and SI. It is the first to comprehensively examine the factors underlying the aforementioned relationship among the Chinese medical students, which was poorly understood thus far. It also provides new and different insights into the problem, which might help in the development of comprehensive plans to reduce the related risk factors, with strong theoretical and practical significance. First of all, the school infirmary should pay more attention to the chronic diseases of medical students, increase the subsidies for their treatment, and dynamically monitor the recovery and physical condition of medical students, so as to detect the rapid aggravation of diseases in time. Second, the monitoring of self-reported sleep quality of these students should be strengthened so that relevant health education can be carried out in schools to improve students' awareness of sleep problems and timely medical treatment can be provided when sleep problems occur. Last of all, and most importantly, the mental health of medical students should be paid attention to. Schools should establish psychological consultation rooms to provide free and regular psychological consultation services for at-risk medical students.

## Methods

### Sample selection

This research was conducted on medical college students between October and November 2018 in Shandong province, China. According to the 2018 National Bureau of Statistics data, this region has a population exceeding 100 million, which makes it the second largest in China. The following sample size calculation formula was employed: *N* = $\frac{{u}_{\alpha /2}^{2}\times \pi (1-\pi )}{{\delta }^{ 2}}$(π: expected prevalence)^59^. According to previous studies, the proportion of medical students who scored above 24 on Beck's SI scale was about 12.75%^60^, in this research, π = 0.128, α = 0.05, ${u}_{\alpha /2}$ = 1.96, and δ = 0.0128. By calculating for the 2,617 samples needed, the failure and rejection of interviews were fully taken into account in the research center. The final sample size of the study was 2800.

This study used a cross-sectional design and a multi-stage cluster sampling technique to select medical undergraduates from two medical schools. It covered all 12 medical majors, including clinical medicine, preventive medicine, pharmacy, stomatology, nursing, and rehabilitation medicine. For each major, we selected all grades from the first to the fourth or the fifth, in each of which, a complete class was chosen to partake in the survey. Overall, 2598 respondents answered the questionnaire, including 1162 boys (44.7%) and 1436 girls (55.3%), with an average age of 20.42 years. They were not compensated with academic credits or anything else. Finally, 2800 questionnaires were distributed, with a response rate of 94.5%, and 2646 valid questionnaires were collected.

### Data collection

The data from the medical undergraduates were collected in the classrooms of their respective schools. Prior to the study’s onset, each researcher underwent a strict training process to ensure consistency across the sample. The postgraduate students of the Shandong University explained the research in detail to the medical ones and informed consent was obtained with their signatures; additionally, all students were informed about the confidentiality of their personal information. Under the researchers’ guidance, the students were requested to complete and submit the questionnaires. To obtain comprehensive, accurate, and high-quality data, we excluded those participants who were unwilling to cooperate with the study. In this survey, each participant was instructed to answer a self-reported battery of questionnaires that consisted of the sociodemographic, chronic disease, sleep quality, mental health, and SI information. The sociodemographic section gathered the data regarding age, gender, grade, region, family composition, parental relationship, and family economic status. At the end of each survey, the finished questionnaires were carefully assessed by quality supervisors.

### Measurement

#### Measurement of chronic diseases

To determine if a medical student had a chronic illness, the question “Do you have a chronic disease?” was asked; it was answered dichotomously as “yes” or “no”. Chronic illnesses include cardiovascular and cerebrovascular diseases (such as hypertension, coronary heart disease, etc.), metabolic diseases (such as diabetes), mental abnormalities, hereditary diseases, chronic occupational illnesses (silicosis, etc.), chronic tracheitis, emphysema, and other chronic ailments.

As we all know, the general population may not be clear about the types and classification of chronic diseases, and there may be deviation. Therefore, we spent a lot of time on unified training on the main indicators of this study, and strictly and uniformly trained the inquiry skills and the inclusion criteria of patients with chronic diseases. Our investigators through learning and mastering the types of these chronic diseases, then our investigators asked each participant if they have one of the types of these chronic diseases one by one. For example, we asked the same participant: Do you have cardiovascular disease? Such as high blood pressure, arrhythmias, and coronary heart disease. After the participants answered whether they had the disease, we then asked: Do you have a metabolic disease? Examples include diabetes, hypoglycemia, obesity, fatty liver, hyperlipidemia, anorexia, hypercalcemia, hypocalcemia, hyperparathyroidism, hypoparathyroidism, parathyroidoma, osteoporosis, osteomalacia, osteodystrophy, hyperuricemia, and acute and chronic gouty arthritis. And then we'll ask: Do you have chronic tracheitis, emphysema, or any other chronic ailments. Finally, we will ask if you have any other disease besides those mentioned above. Based on participants' responses, our investigators used the ICD-10 disease code to determine whether a disease category was included in the chronic disease category. As long as participants had one or more chronic diseases, the answer to that question was yes.

#### Measurement of self-reported sleep quality

The Pittsburgh Sleep Quality Index (PSQI) is a self-administered questionnaire utilized to evaluate self-reported sleep quality and disturbances over a 30-day period^61^. It contains 19 items categorized into seven components: (1) subjective sleep quality; (2) sleep latency; (3) sleep duration; (4) habitual sleep efficiency; (5) sleep disturbances; (6) use of sleeping medication; and (7) daytime dysfunction. On a three-point Likert scale, each component is equally weighted. PSQI is the sum of the scores of seven components, and the total score ranges from 0 to 21; a higher score indicates a lower quality of sleep. We used the Chinese version of the PSQI, which is highly valid and reliable^62^. The Cronbach’s α of this scale in this study was 0.76.

#### Measurement of mental health

The Kessler Psychological Distress Scale (K10) is a widely used 10-item examination, which is used to detect possible disorders based on anxiety, depression or mental distress^63^ and evaluate individuals’ mental health. For each question, subjects were asked to rate how often they had experienced mental distress on a five-point Likert scale of 1 (never) to 5 (always). The scale mainly focuses on anxiety and depression in the past month (four weeks)^64^. The higher the K10 score, the greater the individuals’ mental health. Prior research has used this questionnaire as an indicator of SI^65^. Moreover, in health risk appraisal surveys and primary care assessment, the high precision of K10 has confirmed it to be especially useful as a broad screening scale for mental illness^66^. The Chinese version of the K10, which we used, has previously demonstrated good reliability and validity in Chinese populations^67^. Moreover, it has been employed in the National Comorbidity Survey (NCS)^68^. The Cronbach’s α of this scale in this survey of this scale was 0.92.

#### Measurement of the lifetime and the past 12-month SI

Two separate questions were asked about the lifetime (“Have you ever seriously considered committing suicide?”) and the past 12-month occurrence of SI (“Have you seriously deliberated on committing suicide in the past 12 months?”). They could be answered with a “yes” or a “no”. The question about the SI was derived from the baseline NCS^69^ and has been used extensively in the assessment of SI^4,40^.

### Statement

This study protocol was approved by the Ethical Committee of Shandong University School of Public Health. Written informed consents clarifying the study purposes, significance, methods, and risks were obtained from each participant of all medical undergraduates. And all methods were carried out in accordance with relevant guidelines and regulations.

### Statistical analyses

SPSS PROCESS macro (v3.5, Andrew F. Hayes) and SPSS 24.0 were used for all statistical analyses^70^. First, descriptive analysis, independent-samples Chi-square and T-test were used to preliminary analyze the research data and the SI prevalence rate. Second, after controlling for the statistically significant background characteristics, chronic diseases, self-reported sleep quality, and mental health were entered into the logistic regression models step-by-step, in order to obtain the adjusted OR and the corresponding class intervals. Finally, we used Model 6 of SPSS PROCESS Macro to test the multiple mediator hypothesized model^70,71^. Ordinary least squares regression is the basis of this method^71^.

### Limitations

This study had several limitations. First, it was cross-sectional in nature; thus, causal relationships between study variables remain unestablished. However, associations among these variables are also based on existing theories and previous research support^14,15,24,34,40,56^. Moreover, this research’s findings can provide a basis for further studies to address similar issues through a longitudinal design and interventional trials. Secondly, the use of self-reported data to measure the lifetime and the past 12-month SI, poor mental health, and self-reported poor sleep quality inevitably leads to the possibility of a subjective bias; it would be interesting to witness additional use of clinical-grade diagnostics and sleep monitoring equipment in the future. Thirdly, the issue of suicide is complex; our study mainly focused on the SI risk factors, giving limited consideration to the relationship between the SI and the attempted and completed suicide. Fourthly, the control variables included in this study were limited, and factors such as adverse experiences and PTSD symptoms factors are not included in this study, which can be considered as control factors in future research. This research was used different scales addressing the variables in different timeframes, which may be explored as a new research direction in the future. Finally, this research is applicable only to Chinese medical students, and future studies should verify its generalizability to other populations.

### Ethics approval and consent to participate

The institutional review board of Shandong University School of Public Health (ref.:20181220) approved the study protocol before data collection. Informed consent was obtained for all medical undergraduates aged 16 and over. All methods were carried out in accordance with relevant guidelines and regulations.

## Conclusions

This study suggested a clear association between individual chronic diseases and SI among medical college students. To remediate this problem, the improvement of self-reported sleep quality and reduction of mental health level is required; further, individual chronic diseases should be treated. Physical diseases, sleep and mental health issues need to be addressed through a multidisciplinary team approach and various delivery systems to prevent SI in the aforementioned population.

## Data Availability

The datasets used and/or analysed during the current study are available from the corresponding author on reasonable request.

## Abbreviations

CI: Confidence interval
CNS: Central nervous system
K 10: Kessler Psychological Distress Scale
MGH: Massachusetts General Hospital
NCS: National Comorbidity Survey
NHC: National Health Commission
OR: Odds ratio
SI: Suicidal ideation
PSQI: Pittsburgh Sleep Quality Index
WHO: World Health Organization
